# Squamous cell carcinoma of the cystic duct: A case report and literature review

**DOI:** 10.1097/MD.0000000000035430

**Published:** 2023-10-13

**Authors:** Hui-Jun Wang, Jun-Jie Lu, Ling-Fang Hao, Hai-Na Li, Na Li, Wei-Hua Zheng, Jun-Jing Zhang

**Affiliations:** a Department of General Surgery, Hohhot First Hospital, Hohhot, China; b Department of Oncology, Hohhot First Hospital, Hohhot, China; c Department of Radiology, Hohhot First Hospital, Hohhot, China; d Department of Pathology, Hohhot First Hospital, Hohhot, China;

**Keywords:** case report, chemotherapy, digestive system surgical procedures, gallbladder neoplasms, squamous cell

## Abstract

**Rationale:**

Pure squamous cell carcinoma (SCC) of the gallbladder is a rare malignant biliary tract tumor predominantly found in the body and neck of the gallbladder. However, its occurrence in the cystic duct is even rarer. Given its rarity, no established guidelines or consensus currently exist regarding the treatment of pure SCC of the gallbladder. We report an unusual case of SCC originating from the cystic duct with the intent of providing insights into the therapeutic approach for this type of malignancy.

**Patient concerns:**

A male patient presented to our hospital with acute cholecystitis. Unexpectedly, imaging revealed gallbladder malignancy.

**Diagnoses:**

Pathologic examination after surgery confirmed SCC of the cystic duct.

**Interventions:**

Despite elevated bilirubin levels, we were able to exclude hilar involvement, enabling radical tumor resection. Intraoperatively, we discovered that the tumor was located in the cystic duct, a site associated with a high likelihood of invasion into neighboring organs. The tumor demonstrated a predominantly exophytic growth pattern, which prompted us to refrain from extending the resection range, thereby striking a balance between complete tumor removal and surgical trauma. We performed liver wedge resection only to ensure a negative resection margin while preserving the anatomical structure to the greatest extent possible. Postoperative recovery was rapid and uncomplicated. Pathological examination confirmed pure SCC, which led us to initiate a regimen of nab-paclitaxel and cisplatin, which is known to be effective in other organ SCCs. Remarkably, the patient experienced a rare and severe posttreatment cardiovascular event. Consequently, we switched the patient to a chemotherapy regimen of gemcitabine and cisplatin, which ultimately yielded positive clinical outcomes.

**Outcomes:**

no evidence of tumor recurrence was observed within 1 year after surgery.

**Lessons:**

The diagnosis and therapeutic strategy for rare tumors such as gallbladder SCC should be meticulously tailored based on their unique characteristics to optimize postoperative patient outcomes.

## 1. Introduction

Gallbladder carcinoma is a prevalent malignancy within the biliary tract and notorious for its unfavorable survival prognosis. While adenocarcinoma is the most frequently observed histological subtype of gallbladder carcinoma, squamous cell carcinoma (SCC) is extremely rare, accounting for only 1% to 4% of all gallbladder cancers.^[1]^ SCC rarely originates in the cystic duct. The literature addressing pure SCC of the cystic duct is scarce, attributable to its rarity; as a result, there is a conspicuous absence of pertinent guidelines or consensus. Here, we report a case of pure squamous carcinoma of the cystic duct. Despite a convoluted treatment journey, the patient ultimately had a favorable prognosis. In addition, we provide a review of the current literature on this topic.

## 2. Case presentation

**Figure 1.**
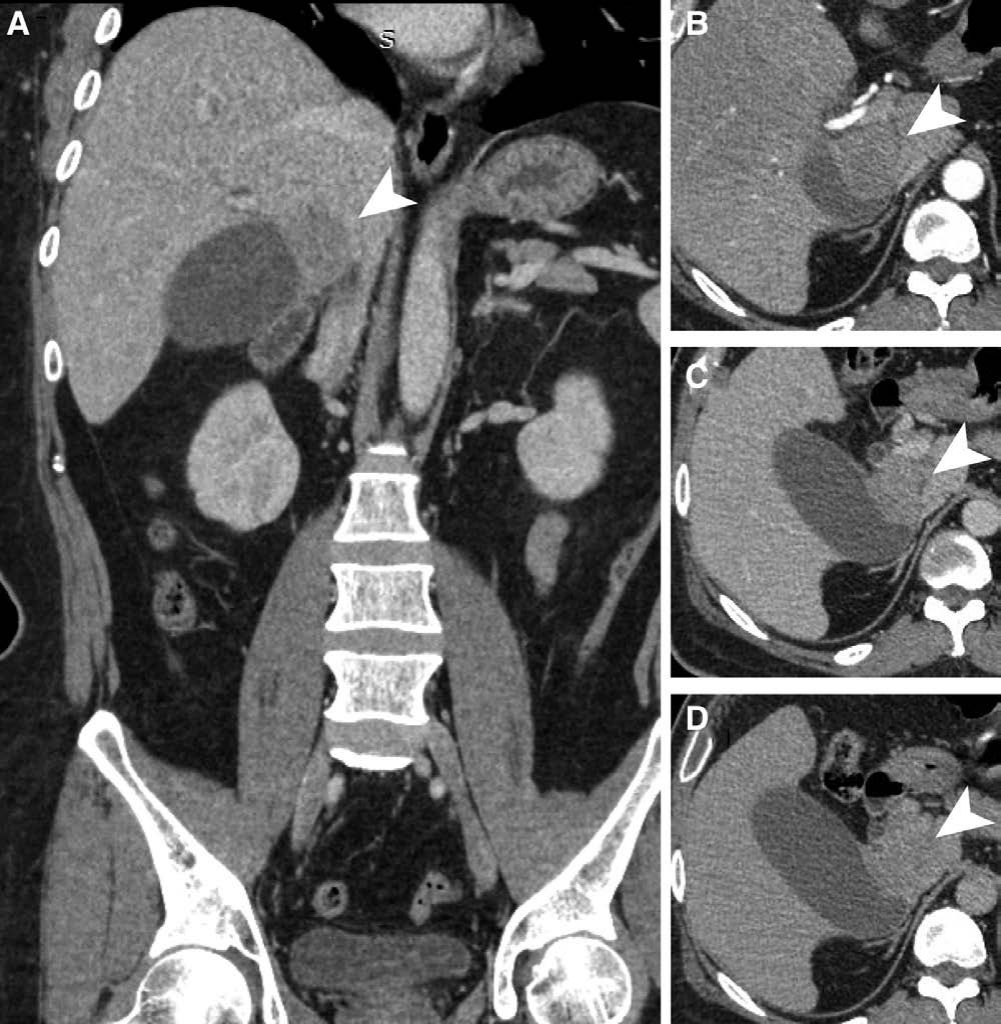
Multiphase contrast-enhanced CT scans. (A) A solid mass in the cystic duct on the coronal plane; (B–D) cross-sectional contrast-enhanced CT shows that the mass was unevenly enhanced, with hypodense shadows that persisted until the delayed phase: (B) arterial phase, (C) venous phase, and (D) delayed phase. Arrows indicate the mass.

A 46-year-old male patient presented with complaints of right upper quadrant pain accompanied by nausea and vomiting, persisting for the preceding 12 hours. His medical history was significant for hypertension, spanning 5 years, and type 2 diabetes in the past year. On physical examination, there was evidence of jaundice, as indicated by yellow staining of the skin and sclera along with a positive Murphy sign. Subsequent blood tests revealed elevated values as follows: white blood cell count, 11.14 × 10^9/L, neutrophil-to-lymphocyte ratio, 79.50%; alkaline phosphatase, 553.00 U/L, total bilirubin, 62.00 μmol/L, and direct bilirubin, 52.00 μmol/L. Tumor marker testing revealed elevated CA12-5 (134.3 U/mL) and CA19-9 (53.41 U/mL). Chest radiography revealed no conspicuous abnormality. However, color Doppler ultrasonography revealed cholestasis, acute cholecystitis, and gallstone incarceration. Abdominal contrast-enhanced CT, in addition to cholecystolithiasis, revealed an irregular soft-tissue mass measuring 57 × 35 mm at the neck of the gallbladder. Notably, the mass demonstrated uneven enhancement, with hypodense shadows persisting into the delayed phase (Fig. 1), and maintained close association with the caudate lobe of the liver, common bile duct, and portal vein. Abnormal enhancement of the lymph nodes posterior to the pancreas suggests possible local metastasis of the gallbladder cancer. These findings were corroborated by abdominal MRI.

**Figure 2.**
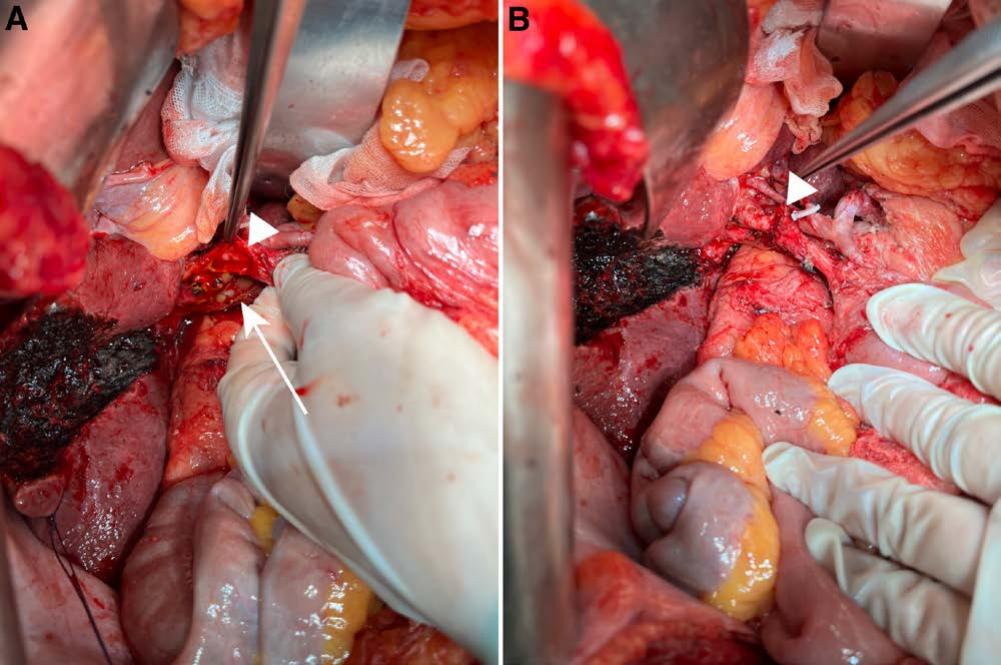
Intraoperative findings. (A) The arrow indicates the wedge resection of a part of portal vein and the anastomosis, and the triangle indicates the wedge resection of the bile duct and (B) The triangle indicates the direct anastomosis after bile duct wedge resection.

Considering the patient’s young age and satisfactory overall health, a decision was made to perform radical resection for gallbladder cancer. Following routine preoperative preparation, abdominal exploration was performed using a right subcostal reversed L-shaped incision. This revealed a mass in the cystic duct and notable swelling of the pancreas and peripancreatic lymph nodes, with no visible evidence of metastasis elsewhere. The hepatoduodenal ligament was skeletonized first, and then lymph node stations 7, 8, 9, 12, 13a, and 16 were dissected. After complete exposure of the hepatic artery, portal vein, and extrahepatic bile duct (except the pancreatic segment), the mass was located on 1 side of the cystic duct at the confluence of the cystic duct and the common hepatic duct, exerting pressure on the inferior side of the left and right bifurcations of the portal vein. The liver parenchyma was resected 2 cm from the liver side of the gallbladder bed and the vessels in the right posterior lobe of the liver were fully preserved. The caudate process involving the caudate lobe was excised because of its close association with the mass. After wedge resection was performed on parts of the portal vein and bile duct, the lateral wall of the portal vein was repaired, and end-to-end anastomosis of the bile duct was performed (Fig. 2). Rapid pathological examination of intra-operative frozen sections showed that the surgical margins were negative.

**Figure 3.**
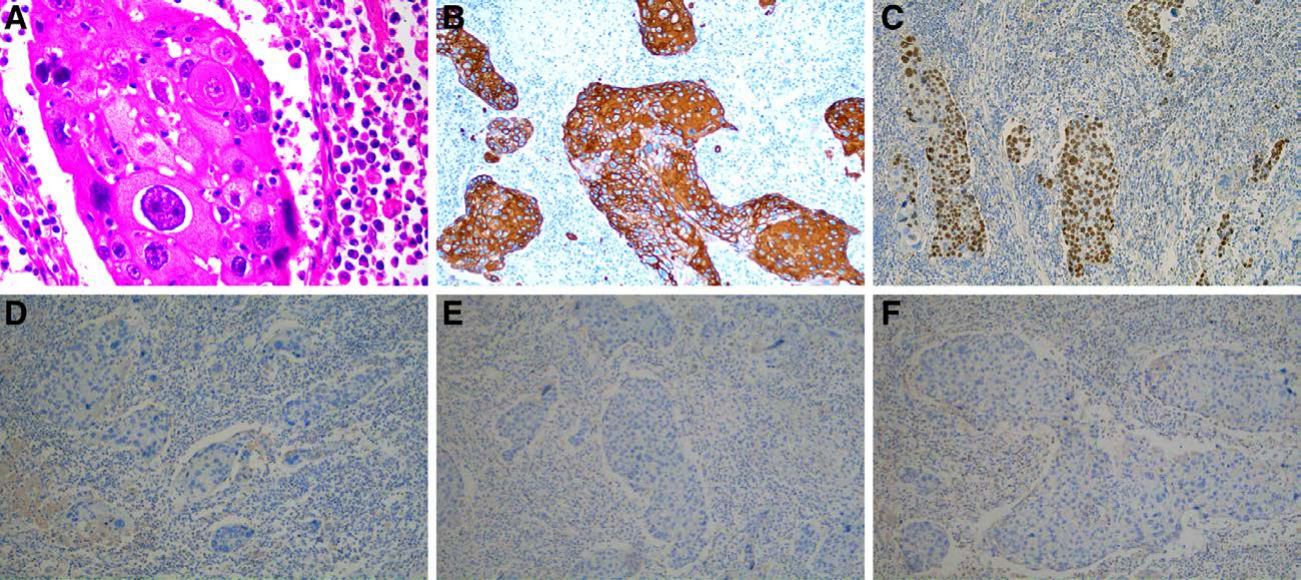
Pathological examination after resection of cystic duct tumor. (A) HE staining (×400). The chromatin was coarsely granular, with obvious nuclear atypia, giant cells, and incomplete keratinization, which are typical features of SCC. (B–F) Immunohistochemical analysis (×40): (B) CK5/6 (+), (C) P63 (+), (D) CK20 (−), (E) SYN (−), and (F) CD56 (−). Positive CK5/6 and P63 suggest squamous cell-derived keratin; CD20 (−), CD56 (−), and SYN (−) exclude adenomatous component and neuroendocrine sources. SCC = squamous cell carcinoma.

The resected specimen showed a mass measuring 40 mm × 40 mm × 38 mm in the cystic duct, and the immunohistochemical findings supported the diagnosis of moderately differentiated pure SCC (Fig. 3). The tumor infiltrated all layers of the gallbladder wall, but did not invade the adjacent liver tissue. Vascular tumor thrombus was observed, and no cancer cells were found on the resection margin. Metastases were detected at lymph node stations 12 and 13 (T_2_N_1_M_0_, IIIB stage). The immunohistochemistry results were CK5/6 (+), P63 (+), CD20 (−), CD56 (−), and SYN (−). There were no postoperative complications, and the patient was able to resume oral feeding 3 days post-operation and was discharged on the 11th postoperative day.

**Figure 4.**
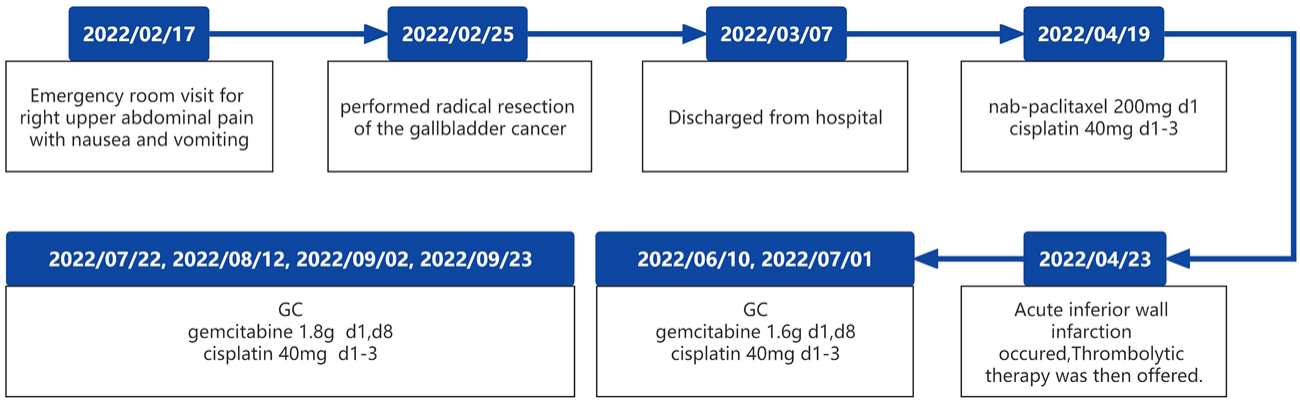
Patient’s clinical history timeline.

One month postoperatively, abdominal CT and MRI revealed no signs of recurrence, and the patient’s overall condition remained satisfactory. Adjuvant chemotherapy was then initiated. The initial regimen consisted of nab-paclitaxel and cisplatin (nab-paclitaxel 200 mg d1 + cisplatin 40 mg d1–3). However, following administration of the first dose, the patient experienced chest pain and extreme sweating. Contrast-enhanced angiography revealed aneurysm-like dilation at the proximal segment of the right coronary artery along with vascular occlusion. We suspected a rare adverse reaction to nab-paclitaxel. Thrombolytic therapy was then initiated. Upon recovery, the chemotherapy regimen was switched to gemcitabine plus cisplatin, repeated every 21 days for a total of 6 cycles (the first 2 cycles of the chemotherapy regimen is gemcitabine 1.6 g d1, d8 + cisplatin 40 mg d1–3 and the last 4 cycles of chemotherapy regimen is gemcitabine 1.8 g d1, d8 + cisplatin 40 mg d1–3). There was no evidence of tumor recurrence within 1 year after surgery. The timeline of all clinical diagnoses and treatments is shown in Figure 4.

## 3. Discussion

Gallbladder malignancies are predominantly adenocarcinomas; the occurrence of pure SCC in the cystic duct is notably infrequent. This rarity has prompted a deeper investigation of the origin, biological properties, and response to treatment. The histological genesis of pure SCC in the gallbladder remains elusive, yet several hypotheses prevail: Metaplasia of pluripotent basal cells in the gallbladder mucosa; Malignant transformation based on squamous metaplasia of the gallbladder mucosa; and Squamous metaplasia based on adenocarcinoma.^[1–3]^ The biological behavior of pure SCC in the gallbladder exhibits unique characteristics. It exhibits a higher local aggressiveness than adenocarcinomas, consequently leading to a higher T stage in patients. Lymphatic spread of pure SCCs has been inconsistently reported in the literature. Leigh et al^[4]^ discovered that the lymph node metastasis rates of pure SCC and adenosquamous carcinoma are significantly higher than those of adenocarcinoma. Conversely, Samuel et al^[3]^ reported the opposite findings, possibly attributed to the amalgamation of pure SCC and adenosquamous carcinoma in the same histological cohort. A recent study by Zhu et al^[2]^ which classified pure SCC and adenosquamous carcinoma into separate groups, found that pure SCC has a lower lymph node metastasis rate than adenocarcinoma. Moreover, the squamous component displays a 2-fold increase in proliferative capacity compared to the adenomatous component, explaining why patients diagnosed with pure SCC tend to have larger tumor volumes.^[2]^

The clinical management of gallbladder adenocarcinoma has largely been established; however, the optimal therapeutic strategy for other rare variant histologies, including pure squamous, adenosquamous, and papillary carcinomas, remains elusive and constrained to institutional series. A lack of consensus regarding the management of these histologies is still evident. Several studies that compared the survival rates of pure SCC and adenocarcinoma following R0 resection concluded that the inherent biological features of pure SCC might be a factor contributing to poor prognosis. Although this conclusion remains controversial,^[5,6]^ it underscores the need for meticulous consideration of radical surgery in frail and elderly populations, given the typically poor outcomes associated with pure SCC.^[1,3,4]^ In patients diagnosed with pure SCC, R0 resection is associated with improved outcomes compared with non-R0 resection. Thus, physically feasible patients are recommended to extend their survival.^[7,8]^ In our case, the patient was otherwise healthy and was not an elderly person. Radical cholecystectomy was performed, and R0 resection was achieved. The patient recovered well after surgery, and no evidence of recurrence was detected in the past 1 year. Notably, the mass was located in the cystic duct, and its invasion into surrounding tissues and organs was larger than that of the tumors of the gallbladder fundus/body. However, in our case, the tumor showed exophytic growth without obvious invasion of the right hepatic artery, the right branch of the portal vein, and the common hepatic duct. The total bilirubin and direct bilirubin levels were significantly elevated in the preoperative examination, which was suspected to be due to tumor invasion of the hepatic hilum; however, fortunately, it was considered to be due to inflammation after careful evaluation by imaging. Some authors have suggested that patients with cystic duct cancer of stage T2 and above should undergo hepatic segmentectomy or even right hemihepatectomy.^[9,10]^ However, in our opinion, resecting an extra centimeter or 2 of the liver is unlikely to alter the natural history of the disease. A negative resection margin will be adequate, and further resection will increase the incidence of postoperative complications.^[11]^ Therefore, a wedge liver resection was performed. Intraoperatively, the mass was found to be closely related to the common hepatic duct and main portal vein, and R0 resection was achieved without altering the anatomy or increasing the risk of tumor recurrence. Wedge-shaped resection of the common hepatic duct and portal vein, end-to-end anastomosis of the common hepatic duct, and repair of the lateral wall of the portal vein were performed, and the patient recovered well after surgery.

Although there is little evidence supporting the role of adjuvant chemotherapy in the treatment of biliary tract cancer (including gallbladder cancer) and data from 3 randomized phase III studies (BCAT, PRODIGE-12/ACCORD-18, and BILCAP) are inconsistent,^[12–14]^ the latest National Comprehensive Cancer Network guidelines support the use of adjuvant chemotherapy for biliary tract cancer, regardless of biliary tract cancer R0/R1 and N0/N1 status. In our case, the tumor was located inside the cystic duct along with lymph node metastases. According to the literature, the presence of lymph node or distant metastasis in patients with cystic duct carcinoma has a strong adverse effect on survival, and these patients are potential candidates for adjuvant chemotherapy.^[15]^ Therefore, adjuvant chemotherapy was recommended. Conventional chemotherapy drugs that can benefit patients with biliary tract cancer may be more feasible for adenocarcinoma, which is the most frequent histological type, and different subtypes respond differently to these chemotherapy drugs.^[16]^ In our patient with pure SCC, nab-paclitaxel plus cisplatin was attempted, as this regimen had been effective in treating SCC of other organs in the past.^[17–20]^ Unexpectedly, the patient developed an acute inferior wall infarction 5 days after the first administration. We concluded that the reason might be related to the aneurysmal dilatation of the right proximal coronary artery combined with the hypercoagulable state of the blood. Considering that the rare severe cardiovascular event might have been caused by nab-paclitaxel, we had to change the chemotherapy regimen for safety reasons. The latest American Society of Clinical Oncology guidelines recommend capecitabine as the standard adjuvant chemotherapy for biliary tract cancer based on the BILCAP study.^[21]^ An intention-to-treat analysis of the BILCAP study did not demonstrate the efficacy of capecitabine, whereas the ABC02 trial revealed that the gemcitabine and cisplatin (GC) regimen had a comparable survival advantage in all subgroups of patients treated with therapeutic chemotherapy regardless of the origin of the biliary tract tumor, suggesting that all patients with biliary tract cancer may benefit from this combination. In addition, some small case series have demonstrated the safety and efficacy of GC regimens in adjuvant settings.^[22,23]^ Therefore, we changed the adjuvant chemotherapy regimen to a GC regimen. To date, the patient has completed all cycles of chemotherapy without any obvious adverse reactions or tumor recurrence. To our knowledge, this is the first report on the use of GC as an adjuvant chemotherapy regimen in patients with pure SCC of the gallbladder. A large multinational study (ACTICCA-1) evaluated GC as an adjuvant chemotherapy combination, and the results are expected to be published in the coming years. Before their data become available, our case report may provide information on the feasibility and effectiveness of GC as an adjuvant chemotherapy for gallbladder cancer, especially for pure SCC of the gallbladder.

## 4. Conclusion

In conclusion, pure SCC of the cystic duct is rare. Similar to many other histological types of gallbladder carcinomas, it presents with nonspecific clinical symptoms, rendering early diagnosis challenging. Vigilance during auxiliary examinations, particularly imaging, is valuable. However, the biological characteristics, treatment options, and prognosis of this rare histological type in unusual locations differ significantly from those of typical gallbladder adenocarcinomas. We would like to highlight the need for surgeons to pay special attention to this variability when formulating a treatment plan instead of directly implementing the treatment plan used for gallbladder adenocarcinoma. A more tailored management approach requires further analysis and an increase in the collection of relevant cases.

## Author contributions

**Conceptualization:** Jun-Jie Lu, Jun-Jing Zhang.

**Formal analysis:** Hui-Jun Wang, Ling-Fang Hao, Wei-Hua Zheng.

**Methodology:** Jun-Jie Lu, Jun-Jing Zhang.

**Resources:** Ling-Fang Hao, Hai-Na Li, Na Li.

**Validation:** Hui-Jun Wang, Jun-Jie Lu, Ling-Fang Hao, Hai-Na Li, Na Li, Wei-Hua Zheng, Jun-Jing Zhang.

**Writing – original draft:** Hui-Jun Wang, Jun-Jie Lu, Wei-Hua Zheng.

**Writing – review & editing:** Hui-Jun Wang, Jun-Jing Zhang.
